# Clinical and psychosocial variables associated to higher costs in schizophrenia spectrum disorders

**DOI:** 10.1192/j.eurpsy.2025.404

**Published:** 2025-08-26

**Authors:** I. Calzavara-Pinton, L. Bertoni, L. Altieri, D. Zardini, N. Necchini, S. Paolini, A. Baglioni, A. Cicale, M. Italia, G. Nibbio, S. Barlati, A. Vita

**Affiliations:** 1 Department of Biomedical Sciences and Translational Medicine, University of Brescia; 2 Department of Mental Health, ASST Spedali Civili di Brescia; 3 Department of Clinical and Experimental Sciences, University of Brescia, Brescia, Italy

## Abstract

**Introduction:**

In Italy, in 2000, the estimated annual economic burden of schizophrenia was 25.000 € per patient, of which 30% were direct costs and 70% indirect costs (Tarricone et al., 2000). Yet, a steep growth has been observed throughout the years: a study showed a yearly expenditure of 41.290 € per patient in 2020 (Latorre et al., 2022).

**Objectives:**

The aim of this study was to better characterize the association between direct costs and clinical and psychosocial variables in schizophrenia spectrum disorders (SSD).

**Methods:**

A total of 276 individuals with schizophrenia spectrum disorders receiving treatment from the Community Mental Health Centers of Brescia (Italy) were included in the study: for each participant socio-demographic, clinical and functional characteristics were assessed, and data related to the use of services in 2022 (then converted to costs) were collected. Clinical and functional characteristics were assessed using the Clinical Global Impression-Severity (CGI-S) scale, the Personal and Social Performance Scale (PSP) scale and the Positive and Negative Syndrome Scale (PANSS). Correlations between the included variables were performed using SPSS v28; values of p <0.05 were considered statistically significant.

**Results:**

Our analyses identified a direct healthcare expenditure of 16477.23 € per patient per year. A positive correlation was observed between higher costs and higher scores at the CGI-S (p<0.001), the PANSS total (p<0.001) and all the PANSS subscales (all p<0.001). Moreover, a negative correlation between higher costs and age of onset (p=0.010) and PSP total score (p<0.001), were observed.

**Conclusions:**

An earlier age of onset, a more severe clinical presentation and a worse psychosocial functioning are associated to a higher expenditure in terms of direct costs associated to use of services in SSD. These results prompt to the implementation of interventions that aim at improving not only clinical aspects, but also functional ones: a full functional recovery would not only benefit patients, but also lead to a lower impact of SSD on healthcare systems. One limitation of the present study is that the estimation of the costs was based on a direct analysis of costs related to the use of services, therefore excluding indirect costs. Future studies should include data on pharmacological treatments, comorbidities and other clinical variables central to the disorder, such as cognition.

**Disclosure of Interest:**

None Declared

